# The impact of a multispecialty operative team on colorectal cancer surgery: A retrospective study from a would-be medical center in Taiwan

**DOI:** 10.1097/MD.0000000000029863

**Published:** 2022-08-05

**Authors:** Chih-I Chen, Fu-Cheng Chuang, Hung-Ju Li, Yu-Chi Chen, Hsin-Pao Chen, Kuang-Wen Liu, Yu-Chieh Su, Jian-Han Chen, Hui-Ming Lee

**Affiliations:** a Division of Colon and Rectal Surgery, Department of Surgery, E-Da Hospital, Kaohsiung, Taiwan; b Division of General Medicine Surgery, Department of Surgery, E-Da Hospital, Kaohsiung, Taiwan; c School of Medicine, College of Medicine, I-Shou University, Kaohsiung, Taiwan; d Department of Information Engineering, I-Shou University, Kaohsiung, Taiwan; e School of Chinese Medicine for Post Baccalaureate, I-Shou University, Kaohsiung, Taiwan; f Department of Radiation Oncology, E-Da Hospital, Kaohsiung, Taiwan; g Division of Hematology-Oncology, Department of Internal Medicine, E-Da Hospital, Kaohsiung, Taiwan; h Department of Urology, E-Da Cancer Hospital, Kaohsiung, Taiwan; i Bariatric and Metabolic International Surgery Center, E-Da Hospital, Kaohsiung, Taiwan; j Division of General Surgery, Department of Surgery, E-Da Hospital, Kaohsiung, Taiwan.

**Keywords:** colon cancer, combined surgery, multidisciplinary surgical team

## Abstract

Some studies showed that when distant metastasis or locally advanced tumors were observed, the participation of 2 or more operating surgeons (combined surgery) in the operation could improve the prognosis of patients. The multispecialty operative team would perform combined surgery in colon cancer patients with some complications since 2015.

The goal of this study is to confirm performing combined surgery would improve the outcomes of colon cancer patients.

A retrospective observational study was conducted, which involved all colon cancer patients between November 2015 and December 2019 at one would-be medical center. Patients were divided into 3 cohorts: those with complicated cases and had combined surgery (C_2S), those with complicated cases and had surgery performed by a single surgeon (C_1S), and those with uncomplicated cases and had surgery performed by a single surgeon (NC_1S). Overall survival and disease-free survival were compared among the 3 groups.

A total of 296 colon cancer patients during the study period. Among them, 35 were C_2S, 87 were C_1S, and 174 were NC_1S. Patients in the NC_1S group had significantly higher 12-, 24-, and 36-month OS rates compared to those in the C_1S group (*P* < .01). In contrast, there was no significant difference in overall survival among patients in the NC_1S and C_2S group (*P* =.15).

The quality of surgery must be impact the prognosis, especially in the individual who was complicated case, the survival in patients who had surgery performed by multispecialty operative team would be improved.

## 1. Introduction

Colorectal cancer (CRC) is the third most commonly diagnosed cancer and the second leading cause of cancer deaths worldwide.^[1]^ In Taiwan, CRC ranked first and second in terms of incidence, but third and fourth in terms of cancer deaths among men and women, respectively. In 2018, 9512 and 7013 new CRC cases were diagnosed among men and women, respectively. The incidence rates were 66.97 and 41.87 per 100,000 persons and the mortality rates were 17.19 and 10.85 per 100,000 persons for men and women, respectively.^[2]^

Since distant metastasis and local recurrence were the most common causes of CRC deaths, the 5-year survival rate of CRC decreased from 74% in stage II to 12% in stage IV between 2013 and 2017. Some studies showed that when distant metastasis or locally advanced tumors were observed, the participation of 2 or more operating surgeons (combined surgery) in the operation could improve the prognosis and outcome of patients.^[3–6]^ In such patients, a multidisciplinary team was reported to offer longer survival after surgery.^[7–9]^ Therefore, since 2015, we had established our multispecialty operative team. The members of our surgical team include specialists such as colorectal surgeons, general surgeons, urologists, gynecologists, and cardiovascular surgeons. All of them are attending physicians and have >5 years of experience in oncologic surgeries. The multispecialty operative team would perform combined surgery in the following complicated cases: clinical staging was T4a, T4b, and M1a; preoperative cystoscopy positive findings; hydronephrosis or hydroureter was detected by preoperative computed tomography (CT) or sonography; suspected metastasis to the ovary or uterus by preoperative CT; and detection of other organ metastases during surgery.

This study aimed to confirm whether surgery performed by the multispecialty operative team on the aforementioned complicated cases improved the survival and other outcomes of patients using the database at our institution. However, because of differences in colon (ICD-O-3 codes: C18.0-C18.9) and rectal cancer (ICD-O-3 codes: C19.9-C20.9), we only focused on colon cancer patients based on our retrospective data to stratify data.

## 2. Methods

### 2.1. Data sources

This retrospective observational study included all colon cancer patients who underwent most definite surgical resection of the primary site between November 1, 2015, and December 31, 2019, at E-Da Hospital. This hospital is a would-be medical center in Kaohsiung, Taiwan, with 900 beds in 2006 and 1259 beds after 2020. Data for this study were obtained from administrative sources and linked with the Taiwan Cancer Registry Database (TCRD). The TCRD provides demographic data, including sex, age at diagnosis, date of birth, as well as cancer-specific information, including primary site, date of most definite surgical resection of the primary site, histology, clinical staging, pathologic staging, date of first recurrence, type of first recurrence, date of last contact or death, vital status, cancer status, and cause of death. All information on the primary cancer site and histology were coded according to the third edition of the International Classification of Diseases for Oncology (ICD-O-3).^[10]^ Each colon cancer stage was based on the sixth edition of the AJCC TNM classification system.^[11]^

### 2.2. Ethics statement

This study protocol was reviewed and approved by the Institutional Review Board of E-Da Hospital and was conducted in accordance with the Helsinki Declaration.

### 2.3. Study design

A total of 418 colon cancer patients underwent surgical resection for the primary site during the study period. Among them, 303 patients had stage II, III, and IVA disease. One patient was excluded due to double cancer and 6 patients were excluded due to neoadjuvant chemotherapy.

### 2.4. Definition of a complicated case

Complicated cases were defined as follows: clinical staging was T4a, T4b, and M1a; preoperative cystoscopy positive findings; hydronephrosis or hydroureter detected by preoperative CT or sonography; suspected metastasis to the ovary or uterus by preoperative CT; and other organ metastasis detected during surgery.

The included 296 patients were divided into 3 groups according to whether the case was complicated or not, and whether the individual’s surgery was performed by 1, 2, or >2 surgeons (combined surgery): those with complicated cases and had combined surgery (C_2S); those with complicated cases and had surgery performed by a single surgeon (C_1S); and those with uncomplicated cases and had surgery performed by a single surgeon (NC_1S). Complete follow-up data were available for all patients.

### 2.5. Outcomes of interest

Overall survival (OS), disease-free survival (DFS), and length of stay (LOS) were the outcomes of interest in our study. OS was defined as the time from the date of the most definite surgical resection of the primary site to the date of last contact or death due to any cause. Patients who were alive or lost to follow up will be censored for OS at the date of last contact. DFS was defined as the time from the date of the most definite surgical resection of the primary site to the date of first recurrence or death due to any cause. LOS was defined as the time from the date of the most definite surgical resection of the primary site to the date of discharge or death.

### 2.6. Statistical analyses

Descriptive analyses of the quantitative data and patient characteristics were performed. Continuous variables were reported as mean, standard deviation (SD), and 95% confidence interval (CI), and compared using analysis of variance. Categorical variables are summarized as numbers and percentages and compared using the chi-square test. Survival curves were plotted and compared using the Kaplan–Meier method and log-rank test. Statistical significance was set at *P* < .05. All statistical analyses were performed using SPSS (version 24.0; IBM Corp. 2016. IBM SPSS Statistics for Windows, version 24.0; IBM Corp. Armonk, NY).

## 3. Results

A total of 296 patients with stage II, III, and IVA colon cancer underwent surgical resection at the primary site between November 2015 and December 2019. Among them, 35 (11.8%) were C_2S, 87 (29.4%) were C_1S, and 174 (58.8%) were NC_1S. Table 1 shows the differences in the clinical characteristics among the 3 groups. There were no significant differences in the sex, age, examined lymph node (ELN), chemotherapy, intestinal obstruction, and surgical mortality (*P* = .12, .52, .29, .90, 0.15, and > .99, respectively). The proportion of patients with stage IVA was significantly higher in the C_2S group (C_2S, 34.3%; C_1S, 26.4%; NC_1S, 0.0%; *P* < .001). However, no significant difference in patients with stage II and stage III disease was observed among the 3 groups (*P* = .67). The percentage of patients receiving targeted therapy was significantly higher in the C_2S group (C_2S, 22.9%; C_1S, 13.8%; NC_1S, 0.6%; *P* < .001). Furthermore, the percentage of patients with bowel perforation was significantly higher in the C_2S group (C_2S, 17.1%; C_1S, 8.0%; NC_1S, 1.7%; *P* < .001).

**Table 1:** Baseline characteristics of the patients.

	C_2S, n = 35	C_1S, n = 87	NC_1S, n = 174	*P*-value
Gender				.12
Male	13 (37.1%)	50 (57.5%)	88 (50.6%)	
Female	22 (62.9%)	37 (42.5%)	86 (49.4%)	
Age				
<65 y old	16 (45.7%)	44 (50.6%)	75 (43.1%)	.52
≥65 y old	19 (54.3%)	43 (49.4%)	99 (56.9%)	
Stage				<.001*
II	11 (31.4%)	29 (33.3%)	90 (51.7%)	
IIa	1 (2.9%)	10 (11.5%)	58 (33.3%)	
IIb	4 (11.4%)	15 (17.2%)	32 (18.4%)	
IIc	6 (17.1%)	4 (4.6%)	0 (0.0%)	
III	12 (34.3%)	35 (40.2%)	84 (48.3%)	
IIIa	0 (0.0%)	1 (1.1%)	12 (6.9%)	
IIIb	5 (14.3%)	18 (20.7%)	51 (29.3%)	
IIIc	7 (20.0%)	16 (18.4%)	21 (12.1%)	
IVA	12 (34.3%)	23 (26.4%)	0 (0.0%)	
ELN				.29
ELN ≥ 12	32 (91.4%)	85 (97.7%)	163 (93.7%)	
ELN < 12	3 (8.6%)	2 (2.3%)	11 (6.3%)	
Chemotherapy				.90
Yes	27 (77.1%)	67 (77.0%)	130 (74.7%)	
No	8 (22.9%)	20 (23.0%)	44 (25.3%)	
Targeted therapy				<.001*
Yes	8 (22.9%)	12 (13.8%)	1 (0.6%)	
No	27 (77.1%)	75 (86.2%)	173 (99.4%)	
Intestinal obstruction				.15
Yes	19 (54.3%)	52 (59.8%)	82 (47.1%)	
No	16 (45.7%)	35 (40.2%)	92 (52.9%)	
Bowel perforation				<.001*
Yes	6 (17.1%)	7 (8.0%)	3 (1.7%)	
No	29 (82.9%)	80 (92.0%)	171 (98.3%)	
Surgical mortality				
Yes	1 (2.9%)	1 (1.1%)	2 (1.1%)	>.99
No	34 (97.1%)	86 (98.9%)	172 (98.9%)	

**Figure 1.**
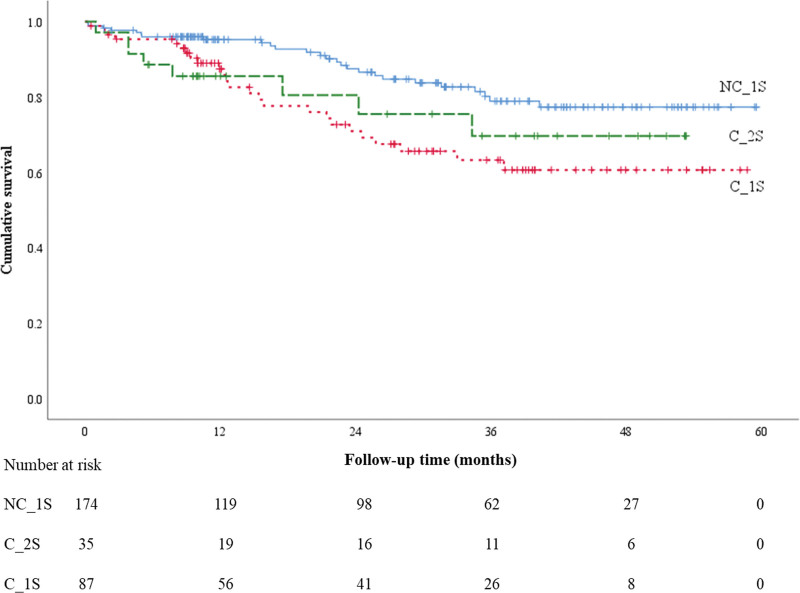
Overall survival.

**Figure 2.**
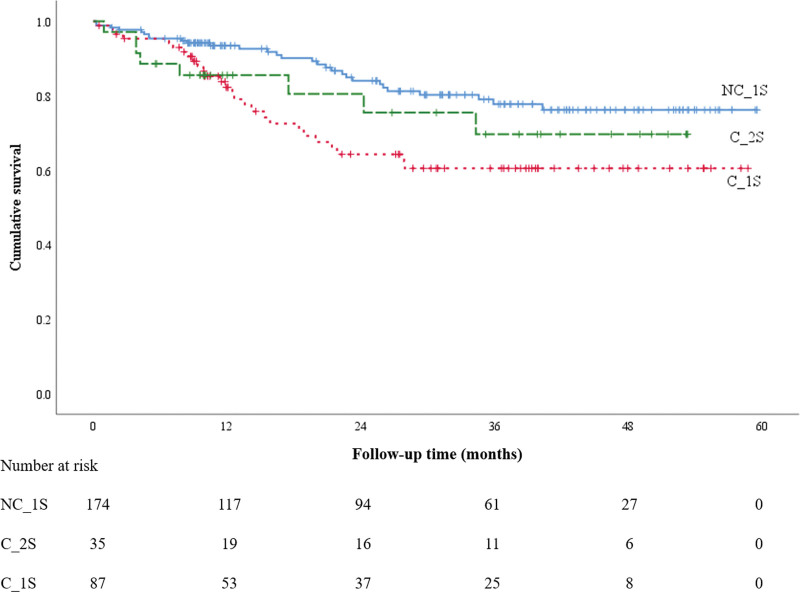
Disease-free survival.

The summary of the OS is presented in Table 2 and Figure 1. Table 2 shows the 12-, 24-, and 36-month OS rates and 95% CIs. The 12-month survival rates of the NC_1S, C_1S, and C_2S groups were 95.2% (95% CI: 92.0%–98.5%), 87.4% (95% CI: 80.1%–94.8%), and 85.5% (95% CI: 73.8%–97.3%), respectively. The 24-month survival rates of the NC_1S, C_1S, and C_2S groups were 87.5% (95% CI: 81.8%–93.1%), 70.9% (95% CI: 60.0%–81.9%), and 80.5% (95% CI: 65.9%–95.1%), respectively. The 36-month survival rates of the NC_1S, C_1S, and C_2S groups were 78.9% (95% CI: 71.2%–86.6%), 63.3% (95% CI: 51.1%–75.4%), and 69.7% (95% CI: 50.7%–88.6%), respectively. Figure 1 presents the Kaplan–Meier survival curves for the patients in the 3 groups. Patients in the NC_1S group had significantly higher 12-, 24-, and 36-month OS rates compared to those in the C_1S group (*P* < .01). In contrast, there was no significant difference in OS among patients in the NC_1S and C_2S groups (*P* = .15). Similarly, a significant difference was observed between the DFS rates of the patients in the NC_1S and C_1S groups (*P* < .01). There was no significant difference in DFS among patients in the NC_1S and C_2S groups (*P* = .25) (Table 3; Fig. 2).

**Table 2:** Summary of overall survival among the 3 groups.

	NC_1S	C_1S	C_2S	
Variable	N = 174	N = 87	N = 35	
No. of deaths (%)	26 (14.9)	25 (28.7)	8 (22.9)	
OS (95% CI)				
12 mo	95.2 (92.0–98.5)	87.4 (80.1–94.8)	85.5 (73.8–97.3)	
24 mo	87.5 (81.8–93.1)	70.9 (60.0–81.9)	80.5 (65.9–95.1)	
36 mo	78.9 (71.2–86.6)	63.3 (51.1–75.4)	69.7 (50.7–88.6)	
		Compared with NC_1S		
*P*	–	<.01*	.15	0.02*

**Table 3:** Summary of disease-free survival among the 3 groups.

	NC_1S	C_1S	C_2S	
Variable	N = 174	N = 87	N = 35	
No. of recur (%)	29 (16.7)	27 (31.0)	8 (22.9)	
DFS (95% CI)				
12 mo	93.4 (89.7–97.2)	82.2 (73.7–90.8)	85.5 (73.8–97.3)	
24 mo	84.0 (77.7–90.3)	64.3 (52.7–75.8)	80.5 (65.9–95.1)	
36 mo	77.7 (70.1–85.3)	60.5 (48.5–72.5)	69.7 (50.7–88.6)	

		Compared with NC_1S		
*P*	–	<.01*	.25	.02*

The 12-month DFS rates were 93.4% (95% CI: 89.7%–97.2%) in the NC_1S group, 82.2% (95% CI: 73.7%–90.8%) in the C_1S group, and 85.5% (95% CI: 73.8%–97.3%) in the C_2S group. The 36-month DFS rates were 77.7% (95% CI: 70.1%–85.3%), 60.5% (95% CI: 48.5%–72.5%), and 69.7% (95% CI: 50.7%–88.6%) in the NC_1S, C_1S, and C_2S groups, respectively.

**Figure 3.**
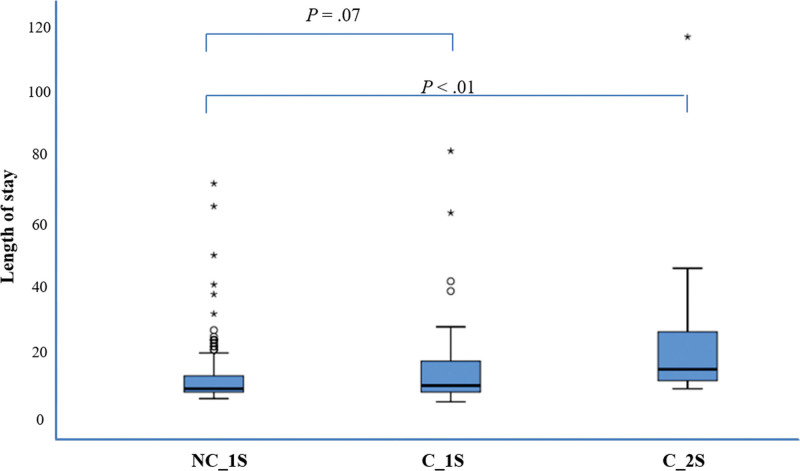
Length of stay.

Figure 3 shows that there was no significant difference in the LOS between the NC_1S and C_1S groups (*P* = .07), but a significant difference between the NC_1S and C_2S groups (*P* < .01) (NC_1S: mean = 12.0 days, SD = 8.9 days, range = 6–72 days; C_1S: mean = 14.3 days, SD = 11.5 days, range = 5–82 days; C_2S: mean = 22.0 days, SD = 19.7 days, range = 9–117 days).

Univariate analysis showed that stage IVA, bowel perforation, and C_1S were associated with a higher risk of mortality, while treatment with chemotherapy was associated with lower mortality (hazard ratio [HR] = 0.315, 95% CI: 0.187–0.531). Patients with bowel perforation had 3.756 times (95% CI: 1.778–9.734) the hazard faced by patients without bowel perforation. The HR of mortality of the patients in the C_1S group significantly increased by 2.169 (*P* < .01) compared with those in NC_1S; however, the HR of the patients in the C_2S group did not significantly increase compared with those in the NC_1S group (HR = 1.800, 95% CI: 0.814–3.977). Mortality was significantly higher in patients with stage IVA (HR = 5.098, 95% CI: 2.361–11.006) and stage III (HR = 2.123, 95% CI: 1.149–3.923) than in patients with stage II (Table 4).

**Table 4:** Univariate analysis of overall survival rate.

	Total (%)	Hazard ratio	95% CI	*P*
Gender				
Male	151 (51.0)	1		
Female	145 (49.0)	0.590	0.348–1.000	.05
Age				
<65 y old	135 (45.6)	1		
≥65 y old	161 (54.4)	1.625	0.953–2.769	.07
Stage				
II	130 (43.9)	1		
III	131 (44.3)	2.123	1.149–3.923	.02*
IVA	35 (11.8)	5.098	2.361–11.006	<.001*
ELN				
ELN < 12	16 (5.4)	1		
ELN ≥ 12	280 (94.6)	0.544	0.217–1.362	.19
Chemotherapy				
No	72 (24.3)	1		
Yes	224 (75.7)	0.315	0.187–0.531	<.001*
Targeted therapy				
No	275 (92.9)	1		
Yes	21 (7.1)	1.660	0.662–4.166	.28
Intestinal obstruction				
No	143 (48.3)	1		
Yes	153 (51.7)	1.261	0.755–2.105	.38
Bowel perforation				
No	280 (94.6)	1		
Yes	16 (5.4)	3.756	1.778–7.934	.001*
Group				
NC_1S	174 (58.8)	1		
C_1S	87 (29.4)	2.169	1.252–3.758	<.01*
C_2S	35 (11.8)	1.800	0.814–3.977	.15

To consider the modeling interaction between the covariates, the groups (C_2S, C_1S, NC_1S), and other covariates were entered in a Cox regression model. The results are presented in Table 5. A significant interaction between the group and age was observed. For patients under 65 years of age, those in the C_1S group had 8.512 times the hazard faced by those in the NC_1S group (95 % CI: 2.813–25.754); however, the HR of mortality of the patients in the C_2S group was not significantly higher than that in the NC_1S group (HR = 2.821, *P* = .23) (Table 6). In addition, there were significant interactions between the group and targeted therapy. For cases without targeted therapy, patients in the C_1S group had 2.2 times the hazard faced by patients in the NC_1S group (95% CI: 1.240–3.904), but the HR of mortality of the patients in the C_2S group was not significantly higher than that in the NC_1S group (HR = 2.067, *P* = .09) (Table 6). However, there was no significant interaction between group and other covariates, including sex, ELN, chemotherapy, intestinal obstruction, and bowel perforation. In addition, a significant effect of gender on mortality was detected in the multivariate model with sex and group. Male sex was associated with higher mortality (HR = 1.729, 95% CI: 1.012–2.955).

**Table 5:** Results for the interaction models and noninteraction models.

Variable	Hazard ratio	*P*	Hazard ratio	*P*
g1	8.509	<.001*		
g2	2.810	.23		
age	4.913	<.01*		
age×g1	0.121	<.01*		
age×g2	0.590	.59		
g1	2.162	.11	2.130	.01*
g2	2.259	.14	2.033	.08
gender	1.807	.15	1.729	.04*
gender×g1	0.976	.97		
gender×g2	0.789	.78		
g1	7.418	.09	2.298	<.01*
g2	0.995	>.99	1.699	.19
ELN	0.455	.20	0.458	.11
ELN×g1	0.302	.32		
ELN×g2	1.901	.60		
g1	3.055	.01*	2.241	<.01*
g2	4.987	.01*	2.111	.07
chem	0.446	.05	0.299	<.001*
chem×g1	0.599	.37		
chem×g2	0.249	.09		
g1	2.202	<.01*		
g2	2.079	.09		
targ	11.518	.02*		
targ×g1	0.106	.06		
targ×g2	0.045	.04*		
g1	3.563	<.01*	2.126	.01*
g2	3.103	.04*	1.792	.15
io	2.001	.09	1.164	.57
io×g1	0.396	.10		
io×g2	0.330	.18		
g1	2.188	.01*	1.963	.02*
g2	1.366	.53	1.484	.34
bp	5.789	.02*	3.070	.01*
bp×g1	0.326	.24		
bp×g2	0.758	.79		

**Table 6:** Results for the stratified models.

Variable	Hazard ratio (95% CI)	*P*
Age < 65, y		
g1	8.512 (2.813–25.754)	<.001*
g2	2.821 (0.516–15.414)	.23
Age ≥ 65, y		
g1	1.028 (0.486–2.171)	.94
g2	1.665 (0.675–4.111)	.27
Without targeted therapy		
g1	2.200 (1.240–3.904)	.01*
g2	2.067 (0.894–4.782)	.09
With targeted therapy		
g1	0.441 (0.043–4.478)	.49
g2	0.132 (0.007–2.664)	.19

## 4. Discussion

In the present study, we found that patients in the NC_1S group had significantly higher 12-, 24-, and 36-month OS rates compared to those in the C_1S group (*P* < .01). In contrast, there was no significant difference in the OS rates among patients in the NC_1S and C_2S groups (*P* = .15). Similarly, a significant difference between the DFS rates of the patients in the NC_1S and C_1S groups was observed (*P* < .01); however, there was no significant difference in the DFS rates among patients in the NC_1S and C_2S groups (*P* = .25). Based on our results, we believe that the survival of patients who underwent surgery performed by a multidisciplinary surgery team would improve.

Surgery plays an important role in the treatment of CRC. Patients who underwent curative resection had a significantly better prognosis.^[12,13]^ Hermanek et al^[12]^ demonstrated that patients who underwent resection for cure (R0) had significantly higher survival rates than those with R1 and R2 resections. The quality of surgery depended on the residual tumor status (categorized by the R classification), the size of the surgical margin, and the ELN number.^[14–16]^ In some special situations, such as locally advanced colon cancer with tumors infiltrating or adherent to the adjacent structures, cT4a or cT4b stage, hydronephrosis, hydroureter, distant metastasis to the ovary or uterus on clinical imaging examinations at diagnosis, and stool per vagina or gas in urine on preoperative examination, we believe that a multidisciplinary surgical team approach should be performed to achieve R0 resection.

The impact of having 2 operating surgeons was demonstrable. Weichman et al^[4]^ compared the outcomes of patients who underwent microsurgical breast reconstruction with single versus 2 operating surgeons and found that the operating room time and hospital LOS decreased significantly. Ames et al^[3]^ showed that the presence of 2 attending surgeons on pedicle subtraction osteotomy decreased the operating time and estimated blood loss. A report comparing outcomes between one versus 2 attending surgeons on adult scoliosis deformity surgery indicated that the co-surgeon approach decreased the incidence of intraoperative complications.^[5]^ In contrast, there were no significant differences in surgery times, hospital LOS, need for revision surgery, or complication rates on a 2-attending approach to microvascular limb reconstruction.^[6]^

E-Da Hospital has been a would-be medical center since 2015 with a multispecialty operative team for CRC treatment established in March 2009. Through intensive cooperation between different specialists and regular weekly group meetings, treatment planning was addressed before the operation. A total of 302 patients with stage II, III, and IVA disease who underwent surgical resection of the primary site were retrospectively reviewed between November 2015 and December 2019.

According to our results, the C_1S group had significantly lower OS rates than those in the NC_1S group. Moreover, the OS rates in the C_2S group was lower than that in the NC_1S group; however, the difference was not statistically significant. This might be because the sample size was not large enough to reveal a difference between the C_2S and NC_1S groups.

Incidentally, in patients with metastatic CRC (mCRC) and complicated cases who had surgery performed by a single surgeon, those treated with targeted therapy had higher OS rates than those treated without targeted therapy. Therefore, targeted therapy should be performed first for mCRC, which is in line with the current treatment guidelines for mCRC,^[17–21]^ giving priority to the treatment of the metastatic site rather than the primary site.

## 5. Limitations

Our study has some limitations. In observational studies, to balance the covariates in the study groups, a propensity score method to generate matched study cohort was used.^[22–25]^ However, the sample size was too small as we performed the matched study. Therefore, a retrospective review of all patients undergoing surgical resection of the primary site between November 2015 and December 2019 was conducted. Selection bias was present. Second, there were several confounding factors in each study group, including clinical staging, the site of distant metastasis, number of metastasis site, adjuvant treatment.^[26]^ The OS of the patients with spontaneous perforation of the cancer was reduced but no statistically significant difference in DFS.^[27]^ Lai et al^[28]^ reported the hypoalbuminemic patients had significantly poorer OS and DFS rates compared to patients with normal serum albumin. Yang et al^[29]^ showed that married patients had better 5-year disease-specific survival compared with unmarried patients, the patients with perineural invasion had lower 5-year disease-specific survival compared with patients without perineural invasion, and the circumferential resection margin distance positively influences survival. Comorbidity was associated with poor short- and long-term survival in CRC patients.^[30]^ Furthermore, the recognition of the circumferential resection margin in locally advanced colon cancer during surgery is different for each surgeon. In clinical T4a or T4b staging, performing R0 resection is often difficult. Thus, it is necessary to further analyze whether the resection margin is malignant.

## 6. Conclusion

According to the retrospective analysis, the quality of surgery affects the prognosis, especially in complicated cases, and the survival of patients who underwent surgery performed by a multispecialty operative team improved. Therefore, for colon cancer patients, surgery performed by the multispecialty operative team could be considered as who clinically staged T4a, T4b, M1a, or had positive findings on preoperative cystoscopy or sonography, hydronephrosis or hydroureter at preoperative CT or sonography, ovarian metastases or uterine metastases evaluated on preoperative CT, and detection of other distant metastases during surgery.

## Acknowledgments

The authors would like to thank Editage (www.editage.com.tw) for the English language editing.

## Author contributions

Conceptualization: Chih-I Chen, Hui-Ming Lee, Yu-Chieh Su

Data curation: Hung-Ju Li

Formal analysis: Hung-Ju Li

Investigation: Fu-Cheng Chuang, Yu-Chi Chen, Hsin-Pao Chen, Kuang-Wen Liu, Yu-Chieh Su, Jian-Han Chen, Hui-Ming Lee

Methodology: Chih-I Chen, Fu-Cheng Chuang

Supervision: Chih-I Chen, Hui-Ming Lee

Validation: Fu-Cheng Chuang, Yu-Chi Chen, Hsin-Pao Chen, Kuang-Wen Liu, Yu-Chieh Su, Jian-Han Chen, Hui-Ming Lee

Visualization: Chih-I Chen, Hung-Ju Li

Writing - original draft: Chih-I Chen, Hung-Ju Li

Writing - review & edition: all authors

## Footnotes

* Statistically significant.

* Statistically significant.

* Statistically significant.

* Statistically significant.

## References

[1] Sung H Ferlay J Siegel RL. Global cancer statistics 2020: GLOBOCAN estimates of incidence and mortality worldwide for 36 cancers in 185 countries. CA: A Cancer J Clin. 2021;71:209–49. 10.3322/caac.21660 33538338

[2] Health Promotion Administration. Ministry of Health and Welfare, Taiwan. Cancer Registry Annual Report. Available at: http://www.hpa.gov.tw/BHPNet/Web/Stat/Statistics.aspx. 2021.

[3] Ames CP Barry JJ Keshavarzi S. Perioperative outcomes and complications of pedicle subtraction osteotomy in cases with single versus two attending surgeons. Spine Deform. 2013;1:51–8. 27927323 10.1016/j.jspd.2012.10.004

[4] Weichman KE Lam G Wilson SC. The impact of two operating surgeons on microsurgical breast reconstruction. Plast Reconstr Surg. 2017;139:277–84. 28121853 10.1097/PRS.0000000000002946

[5] Gomez JA Lafage V Scuibba DM. Adult scoliosis deformity surgery: comparison of outcomes between one versus two attending surgeons. Spine (Phila Pa 1976). 2017;42:992–8. 28098740 10.1097/BRS.0000000000002071

[6] Ehrl D Heidekrueger PI Ninkovic M. Impact of two attendings on the outcomes of microvascular limb reconstruction. J Reconstr Microsurg. 2018;34:59–64. 28973712 10.1055/s-0037-1606541

[7] Lan Y-T Jiang J-K Chang S-C. Improved outcomes of colorectal cancer patients with liver metastases in the era of the multidisciplinary teams. Int J Colorectal Dis. 2016;31:403–11. 26662193 10.1007/s00384-015-2459-4

[8] Rogers MJ Matheson L Garrard B. Comparison of outcomes for cancer patients discussed and not discussed at a multidisciplinary meeting. Public Health. 2017;149:74–80. 28575751 10.1016/j.puhe.2017.04.022

[9] Foucan AS Grosclaude P Bousser V. Management of colon cancer patients: a comprehensive analysis of the absence of multidisciplinary team meetings in two French departments. Clin Res Hepatol Gastroenterol. 2021;45:101413. 32359832 10.1016/j.clinre.2020.02.020

[10] Fritz A Percy C Jack A. International Classification of Diseases for Oncology: ICD-O. 3rd ed. Geneva, Switzerland: World Health Organization, 2000.

[11] Greene FL Balch CM Fleming ID. AJCC Cancer Staging Manual. 6th edn. New York: Springer Verlag, 2002.

[12] Hermanek P Mansmann U Staimmer D. The German experience: the surgeon as a prognostic factor in colon and rectal cancer surgery. Surg Oncol Clin N Am. 2000;9:33–49. 10601523

[13] Wittekind C Compton CC Greene FL. TNM residual tumor classification revisited. Cancer. 2002;94:2511–6. 12015777 10.1002/cncr.10492

[14] Osterman E Glimelius B. Recurrence Risk after up-to-date colon cancer staging, surgery, and pathology: analysis of the entire Swedish population. Dis Colon Rectum. 2018;61:1016–25. 30086050 10.1097/DCR.0000000000001158

[15] Zhu X Zhao M Zhou L. Significance of examined lymph nodes number and metastatic lymph nodes ratio in overall survival and adjuvant treatment decision in resected laryngeal carcinoma. Cancer Med. 2020;9:3006–14. 32112627 10.1002/cam4.2902 PMC7196060

[16] Huang L Jansen L Balavarca Y. Significance of examined lymph node number in accurate staging and long-term survival in resected stage I–II pancreatic cancer—more is better? a large international population-based cohort study. Ann Surg. 2021;274:e554–63. 31425290 10.1097/SLA.0000000000003558

[17] Sánchez-Gundín J Fernández-Carballido AM Martínez-Valdivieso L. New trends in the therapeutic approach to metastatic colorectal cancer. Int J Med Sci. 2018;15:659–65. 29910669 10.7150/ijms.24453 PMC6001415

[18] Xie Y-H Chen Y-X Fang J-Y. Comprehensive review of targeted therapy for colorectal cancer. Signal Transduct Target Ther. 2020;5:22. 32296018 10.1038/s41392-020-0116-z PMC7082344

[19] Bellio H Fumet JD Ghiringhelli F. Targeting BRAF and RAS in colorectal Cancer. Cancers. 2021;13:2201. 34063682 10.3390/cancers13092201 PMC8124706

[20] Shek D Akhuba L Carlino MS. Immune-checkpoint inhibitors for metastatic colorectal cancer: a systematic review of clinical outcomes. Cancers. 2021;13:4345. 34503155 10.3390/cancers13174345 PMC8430485

[21] Catalano F Borea R Puglisi S. Targeting the DNA damage response pathway as a novel therapeutic strategy in colorectal cancer. Cancers. 2022;14:1388. 35326540 10.3390/cancers14061388 PMC8946235

[22] D’Agostino RB Jr. Propensity score methods for bias reduction in the comparison of a treatment to a non-randomized control group. Stat Med. 1998;17:2265–81. 9802183 10.1002/(sici)1097-0258(19981015)17:19<2265::aid-sim918>3.0.co;2-b

[23] Streiner DL Norman GR. The pros and cons of propensity scores. Chest. 2012;142:1380–2. 23208333 10.1378/chest.12-1920

[24] Asaad M Xu Y Chu CK. The impact of co-surgeons on complication rates and healthcare cost in patients undergoing microsurgical breast reconstruction: analysis of 8680 patients. Breast Cancer Res Treat. 2020;184:345–56. 32803638 10.1007/s10549-020-05845-6

[25] Halliday LJ Doganay E Wynter-Blyth VA. The impact of prehabilitation on post-operative outcomes in oesophageal cancer surgery: a propensity score matched comparison. J Gastrointest Surg. 2021;25:2733–41. 33269459 10.1007/s11605-020-04881-3 PMC8602132

[26] Wang J Li S Liu Y. Metastatic patterns and survival outcomes in patients with stage IV colon cancer: a population-based analysis. Cancer Med. 2020;9:361–73. 31693304 10.1002/cam4.2673 PMC6943094

[27] Abdelrazeq AS Scott N Thorn C. The impact of spontaneous tumour perforation on outcome following colon cancer surgery. Colorectal Dis. 2008;10:775–80. 18266887 10.1111/j.1463-1318.2007.01412.x

[28] Lai C-C You J-F Yeh C-Y. Low preoperative serum albumin in colon cancer: a risk factor for poor outcome. Int J Colorectal Dis. 2011;26:473–81. 21190025 10.1007/s00384-010-1113-4

[29] Yang C-C Cheng L-C Lin Y-W. The impact of marital status on survival in patients with surgically treated colon cancer. Medicine. 2019;98:e14856–e14856. 30882684 10.1097/MD.0000000000014856 PMC6426559

[30] Boakye D Rillmann B Walter V. Impact of comorbidity and frailty on prognosis in colorectal cancer patients: a systematic review and meta-analysis. Cancer Treat Rev. 2018;64:30–9. 29459248 10.1016/j.ctrv.2018.02.003

